# Transcriptomic changes in barley leaves induced by alcohol ethoxylates indicate potential pathways of surfactant detoxification

**DOI:** 10.1038/s41598-024-54806-2

**Published:** 2024-02-24

**Authors:** Johanna Baales, Viktoria V. Zeisler-Diehl, Tino Kreszies, Alina Klaus, Frank Hochholdinger, Lukas Schreiber

**Affiliations:** 1 Department of Ecophysiology, Institute of Cellular and Molecular Botany, University of Bonn, Kirschallee 1, 53115 Bonn, Germany; 2 Department of Crop Science, Plant Nutrition and Crop Physiology, University of Göttingen, Carl-Sprengel-Weg 1, 37075 Göttingen, Germany; 3 Institute of Crop Science and Resource Conservation (INRES), Crop Functional Genomics, University of Bonn, 53113 Bonn, Germany

**Keywords:** Biochemistry, Plant sciences

## Abstract

Hardly anything is known regarding the detoxification of surfactants in crop plants, although they are frequently treated with agrochemical formulations. Therefore, we studied transcriptomic changes in barley leaves induced in response to spraying leaf surfaces with two alcohol ethoxylates (AEs). As model surfactants, we selected the monodisperse tetraethylene glycol monododecyl (C_12_E_4_) ether and the polydisperse BrijL4. Barley plants were harvested 8 h after spraying with a 0.1% surfactant solution and changes in gene expression were analysed by RNA-sequencing (RNA-Seq). Gene expression was significantly altered in response to both surfactants. With BrijL4 more genes (9724) were differentially expressed compared to C_12_E_4_ (6197). Gene families showing pronounced up-regulation were cytochrome P450 enzymes, monooxygenases, ABC-transporters, acetyl- and methyl- transferases, glutathione-S-transferases and glycosyltransferases. These specific changes in gene expression and the postulated function of the corresponding enzymes allowed hypothesizing three potential metabolic pathways of AE detoxification in barley leaves. (i) Up-regulation of P450 cytochrome oxidoreductases suggested a degradation of the lipophilic alkyl residue (dodecyl chain) of the AEs by ω- and β- oxidation. (ii) Alternatively, the polar PEG-chain of AEs could be degraded. (iii) Instead of surfactant degradation, a further pathway of detoxification could be the sequestration of AEs into the vacuole or the apoplast (cell wall). Thus, our results show that AEs lead to pronounced changes in the expression of genes coding for proteins potentially being involved in the detoxification of surfactants.

## Introduction

Surfactants are widely used in many industrial and private applications. Thus, it is unavoidable that they also tend to end up in the environment leading for example to the contamination of soil and freshwater ecosystems causing ecotoxicological problems^1^. As a consequence, many surfactants with a low rate of biodegradation are prohibited in the meantime and today highly biodegradable surfactants, such as alcohol ethoxylates (AEs), are preferentially used in many applications^2–4^.

AEs are non-ionic surfactants and they consist of a polar but uncharged head group formed by a varying number of ethylene units (E) and a hydrophobic tail formed by linear or branched hydrocarbon chains of varying length (C). The general molecular formula of AEs is given as C_x_E_y_, with x = chain length of the hydrophobic part and y = number of ethylene oxide units. Physicochemical properties of AEs change with the ethylene oxide content, the chain length and their structure^5,6^. These different properties can influence the function of AEs as well as their degradation^7,8^.

Agrochemical formulations sprayed to leaf surfaces typically contain surfactants since they promote foliar uptake of active ingredients (AIs) in leaves^9^. Surfactants help to enhance leaf surface wetting and spreading of the spray droplets^10,11^. Enhanced wetting is of major importance for many crop species characterized by superhydrophobic leaf surfaces having contact angles for water droplets higher than 140 degree^12,13^. In addition, surfactants induce plasticizing effects on the highly impermeable cuticular transport barrier, which significantly enhances the diffusion of AIs through the cuticle into the plant interior^14^. Thus, it must be postulated that surfactants must be taken up by the leaves in parallel to AIs. However, basically, nothing is known about their fate inside the living plant tissue and whether they are degraded.

Activation of cytochrome P450 enzymes, glycosyl transferases and glutathione transferases are known mechanisms for detoxification of xenobiotics in plants^15–17^. Once xenobiotics have been taken up by the plant and are activated by monooxygenases or peroxidases, conjugated to glutathione or a hexose they might get accumulated in vacuoles, incorporated in the cell, cell wall, or excreted^18^. Enzymes involved in the detoxification are often linked to the regulation of cellular redox state and plant mitochondrial respiratory chain affecting energy status and primary metabolism^19^. Nevertheless, the metabolism of many xenobiotics is still unclear due to possible interference with plant primary- and especially secondary metabolism^20^.

During the last decades, much attention was paid to the bacterial degradation of surfactants in sewage treatment plants and soil^4,21^. Metabolic pathways of AE degradation identified in bacteria include the ω- and β- oxidation of the hydrophobic alkyl chain and cleavage of C_2_ units from the polar ethoxylated part of the AEs^7,8,22^. However, little attention was given to the possible degradation or detoxification of surfactants in crop plants themselves, which are directly exposed to surfactants. Consequently, the question arises whether similar or different metabolic processes are activated in crops frequently exposed to surfactants during spray application of agrochemical formulations^23^.

In a previous study, we showed that AEs sprayed to leaf surfaces of barley plants are rapidly taken up by the leaves within a few hours^24^. At higher concentrations (1% and 10%), leaf photosynthesis was severely affected and leaves were rapidly dying within a couple of hours indicating the toxicity of AEs. Effects on photosynthesis for example can be explained by the fact that surfactants disturb membranes by intercalating within phospholipid bilayers, which in turn leads to changes in membrane fluidity and integrity^25^. However, spraying aqueous AE solutions at realistic concentrations of 0.1% to barley leaf surfaces, as they are used during spray application in the field^26^, did not at all affect leaf physiology (photosynthesis and transpiration). These results indicate that AEs can be toxic to barley leaves when applied at higher concentrations, but apparently not at lower concentrations. Thus, it can be speculated that barley leaves might have the potential of detoxifying AEs by degradation or sequestration. Our RNA-Seq analysis shows that AEs lead to differential gene expression providing first hints for the potential degradation and detoxification of AEs in barley leaves.

## Results

### Transcriptomic analysis using RNA-Seq

**Figure 1:**
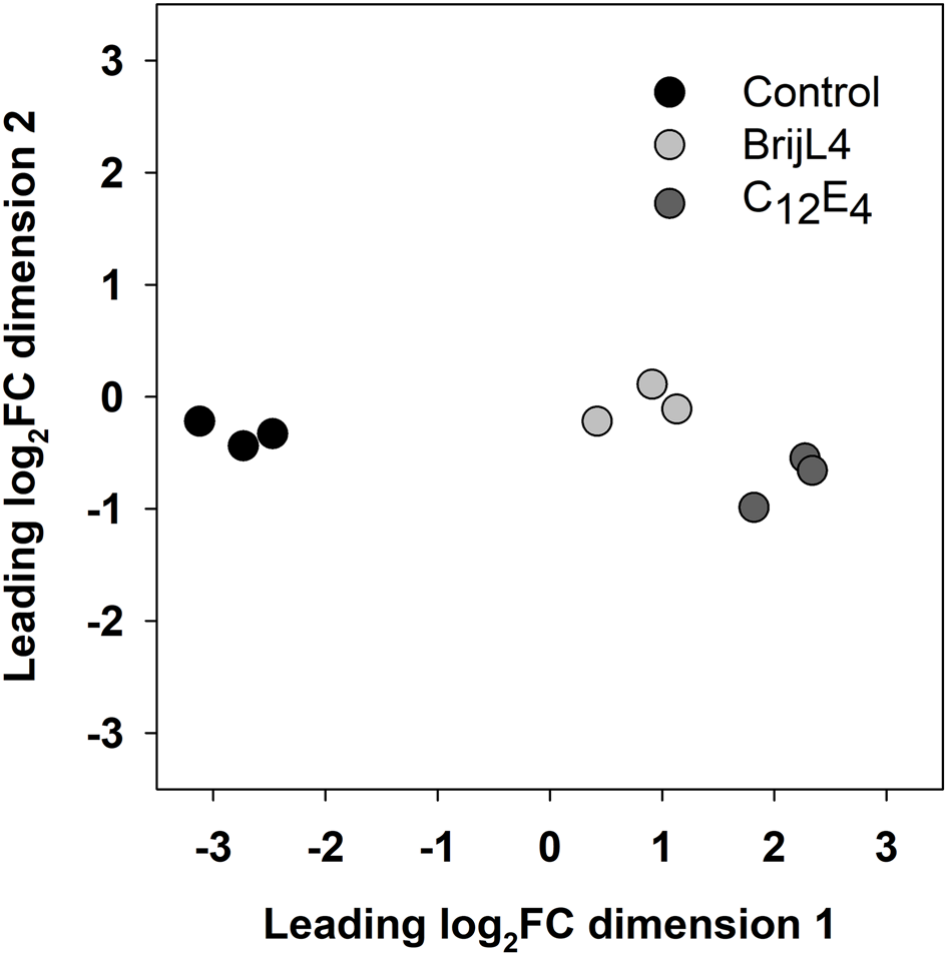
Multidimensional scaling plot of the RNA-Seq samples of controls (black circle), BrijL4-treated leaves (light grey circles) and C_12_E_4_-treated leaves (dark grey circles). Samples of the controls clearly separate from the two treatments with alcohol ethoxylates, which themselves cluster closer together.

**Figure 2:**
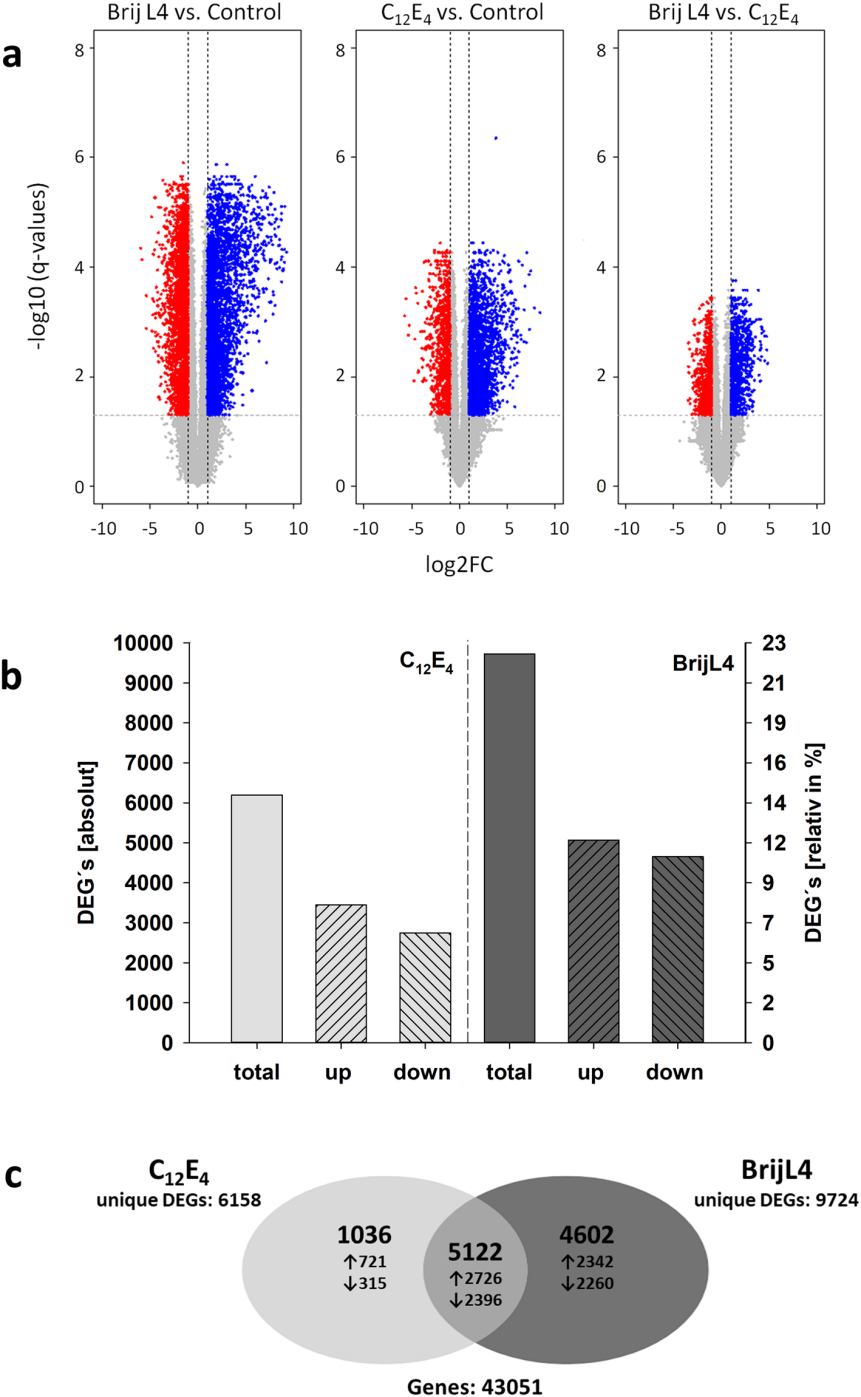
Summary of differentially expressed genes (DEGs) between control and surfactant treatments. (**a**) Volcano plots depict up-regulated (blue dots) and down-regulated DEGs (red dots). DEGs that do not exceed the threshold of log_2_-fold changes │log_2_FC│ > 1 and false discovery rates (FDR) log_10_ (q-values) ≤ 1% are depicted in grey. Venn diagram (**b**) indicating the overlap between DEGs after treatment with C_12_E_4_ (light grey) or BrijL4 (dark grey). Arrows indicate number of up- or down-regulated DEGs.

RNA-Sequencing (RNA-Seq) yielded on average 35 million reads for each sample. The multidimensional scaling (MDS) plot shows that the three replicates of each treatment cluster closely together while the control and the two AEs (C_12_E_4_ vs. BrijL4) treatments are clearly separated (Fig. 1). The number of DEGs between control and surfactant treatments (FDR ≤ 1%, |log_2_FC|> 1) are depicted as volcano plots (Fig. 2a) and Venn diagram (Fig. 2b). Overall, the BrijL4 treatment leads to a substantially higher number (total: 9724 DEGs; 23%) of DEGs than the treatment with C_12_E_4_ (total: 6197 DEGs; 14%) (Fig. 2b). In total, 5068 unique genes were up-regulated and 4656 unique genes were down-regulated after treatment with BrijL4 (Fig. 2b). C_12_E_4_ leads to an up-regulation of 3447 and a down-regulation of 2747 unique genes (Fig. 2b). Cross comparison between the two surfactants shows that 2726 genes are up-regulated and 2396 are down-regulated in both treatments.

### Assessment of metabolic pathways used in the degradation of linear alcohol ethoxylates

Functional categorization was performed by identification of significantly enriched GO terms by single enrichment analysis with AgriGOv2^27^. The analysis showed 72 unique enriched GO terms when comparing the DEGs between control and treatment and between C_12_E_4_ treatment and BrijL4 treatment (Table 1). In total, 18 GO terms are up- and 2 are down-regulated in both treatments (Table 1).

**Table 1:**
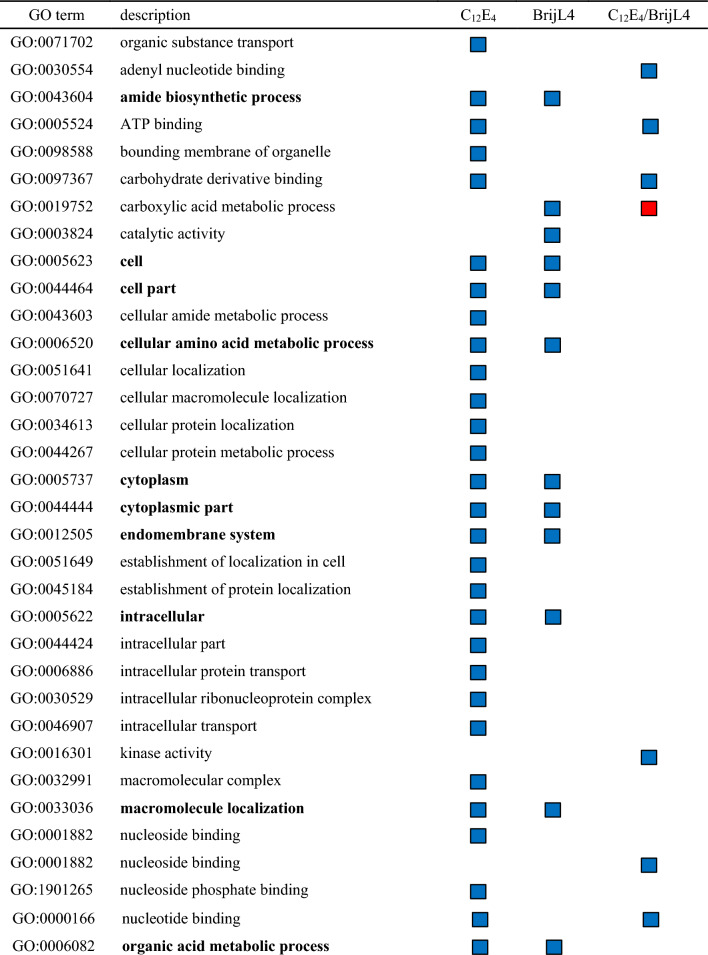

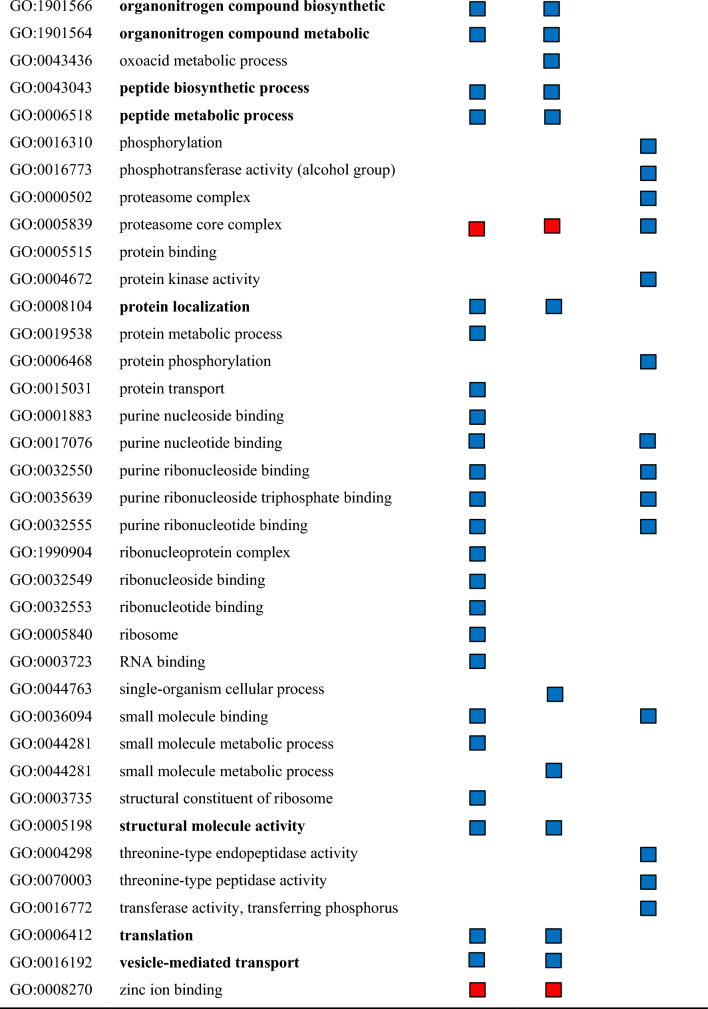
Enriched functional gene ontology (GO) terms among differentially expressed genes (DEGs) in response to C_12_E_4_ or BrijL4 treatments of barley leaves. GO terms given are non-redundant terms (similarity ≤ 0.5) with log_2_-fold changes (log_2_FC) ≤ 1%. The direction of regulation is given in blue squares (up-regulated) and red squares (down-regulated). No symbol indicates not significantly up or down regulated. Go terms showing an up- or down-regulation in both surfactant treatments are highlighted in bold type.

Filtering with REVIGO indicated that significantly enriched biological processes in response to surfactant treatments were (i) biosynthetic and metabolic processes, (ii) transport and localisation and (iii) protein metabolism (Table 2). The biological processes of zinc ion and protein binding are most down-regulated. Moreover, ubiquitin-protein transferase activity, chloride channel activity and binding processes are down-regulated in response to C_12_E_4_ treatment. GO terms assigned to cellular components showed enriched gene expression related to (i) ribosomes, (ii) membranes of organelles and (iii) the proteasome complex after C_12_E_4_ treatment. BrijL4 showed enriched activity at (i) ribosomes, (ii) the cytoplasm and (iii) the endomembrane systems. GO terms assigned to molecular functions showed enriched gene activity related to (i) GTP binding, (ii) small GTPase activity and (iii) catalytic activity after C_12_E_4_ treatment. After BrijL4 treatment GO terms assigned to molecular functions showed enriched gene activity related to (i) GTP binding, (ii) cofactor and molecule binding and (iii) small GTPase activity. In both treatments, the analysis showed a weaker enrichment of gene activity related to (i) ligases, (ii) isomerases and (iii) transferase activity (Table 2).

**Table 2:** Enriched functional gene ontology (GO) terms among differentially expressed genes DEGs responding to treatment of barley leaves with C_12_E_4_ or BrijL4 in relative values (%) calculated using REVIGO (reduce and visualize gene ontology). The gene ontology covers three domains. Biological processes: operation or sets of molecular events with a defined beginning and end. Cellular component: the part of a cell or its extracellular environment. Molecular function: the element activities of a gene product at the molecular level.

	C_12_E_4_	BrijL4
**Biological processes (up regulated)**	[%]	[%]
Biosynthetic and metabolic processes	64	43
Transport and localisation	18	30
Protein metabolism	16	21
Small GTPase medicated signal transduction	–	3
Cellular process	2	3
**Biological processes (down regulated)**		
Zinc ion binding	58	76
Protein binding	15	24
Ubiquitin-protein transferase activity	13	–
Chloride channel activity	7	–
Binding	7	–
**Cellular component**		
Ribosome	54	67
Organelle membrane	10	2
Proteasome complex	9	3
Cytoplasm	7	9
Mitochondria	5	2
Endoplasmic reticulum	5	–
Membrane	5	–
Coated membrane	2	–
Whole membrane	–	3
Endomembrane system	2	4
Golgi apparatus	–	2
Envelope	–	2
Outer membrane	–	1
**Molecular function**		
GTP-binding	27	45
Small GTPase activity	22	11
Catalytic activity	13	–
Cofactor and molecular binding	11	15
Structural constituent of ribosome	10	6
Structural molecule activity	7	–
Ligase activity	5	4
Isomerase activity	5	–
Aminoacyl-tRNA ligase activity	–	4
Transferase activity	–	3
Unfolded protein binding	–	3

The MapMan analyses indicated a high activity of DEGs in response to biotic stress, lipid transfer proteins, photosynthesis, respiration, carbohydrate metabolism and coenzyme metabolism as well as in large enzyme families (Table 3).

**Table 3:**
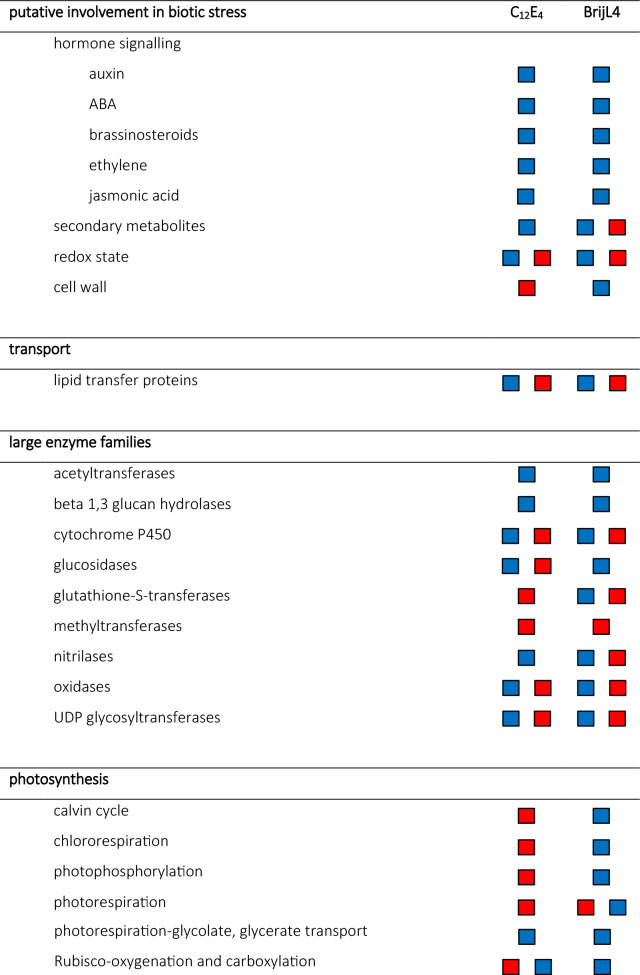

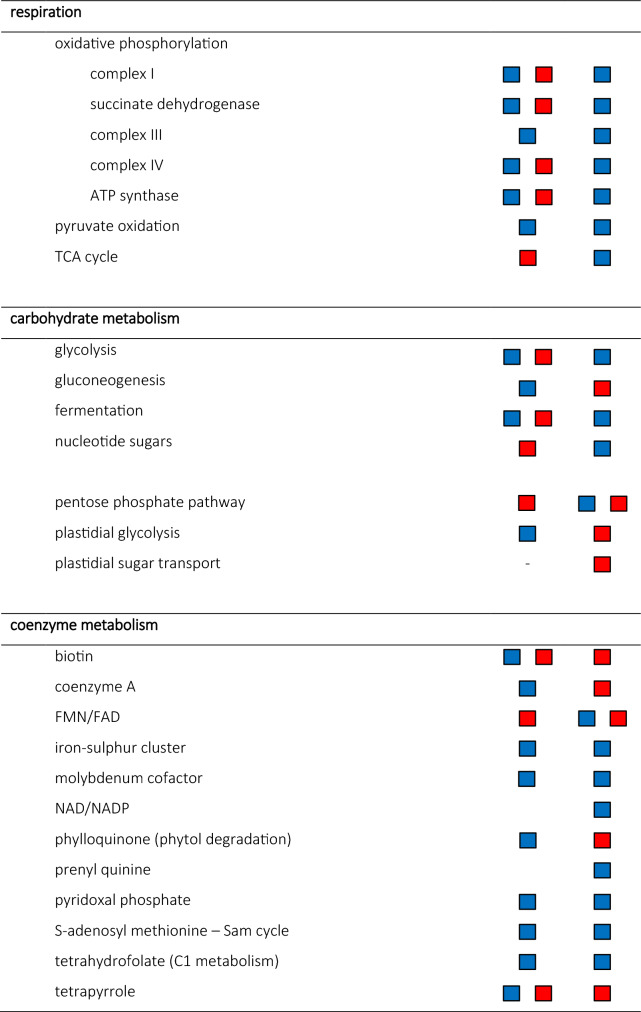
Differentially expressed genes (DEGs) in response to C_12_E_4_ or BrijL4 treatment of barley leaves. DEGs are shown as groups associated with putative involvement in biotic stress, transport, large enzyme families, photosynthesis, respiration, carbohydrate metabolism and coenzyme metabolism based on pre-existing biological knowledge using MapMan and Ensemble Plant. The direction of regulation is given in blue squares (up-regulated) and red squares (down-regulated). No symbol indicates not significantly up or down regulated.

Finally, DEGs highly up-regulated within the RNA-Seq dataset for both treatments were identified via Ensemble Plants and UniProt. Up-regulated genes indicate a general stress response due to treatment with either BrijL4 or C_12_E_4_ (Table 4). In both treatments, genes coding for monooxygenases such as cytochrome P450, glycosyltransferases, glutathione-S-transferases, malonyl-, acetyl-and methyl- transferases, ABC-transporters and oxidases were highly up-regulated.

**Table 4:** Differentially expressed genes (DEGs) up-regulated in response to the treatment of barley leaves with C_12_E_4_ or Brij L4. Gene IDs are given together with log_2_-fold changes (log_2_FC). Putative gene functions were hypothesized by the function of corresponding orthologues genes in *Arabidopsis thaliana* or *Triticum urartu* as described in literature. Small numbers in brackets indicate the postulated steps of degradation/detoxification as described in detail in Fig. 3.

Annotated function	Gene name	Barley ID	Log_2_FC	
			C_12_E_4_	BrijL4
Acetyl transferase	*AT5G07860*	HORVU3Hr1G023780	3.10	4.90
Alcohol dehydrogenase^(2)^	*ADH1*	HORVU4Hr1G016810	2.10	4.23
	*AT1G22430*	HORVU2Hr1G029960	3.23	–
Alcohol oxidase	*FAO1*	HORVU0Hr1G015950	0.88	
Aldehyde dehydrogenase^(4)^	*ALDH3H1*	HORVU4Hr1G017450	0.31	0.38
ATP-binding cassette^(5)^ (ABC) transporter	*ABCB7*	HORVU3Hr1G065320	3.22	5.89
	*WBC1 /ABCG13*	HORVU1Hr1G030200	0.83	0.74
	*WBC11/ABCG11*	HORVU2Hr1G090960	1.24	1.23
	*TRIUR3_02168*	HORVU5Hr1G106850	4.18	6.20
	*TRIUR3_07157*	HORVU3Hr1G024210	4.21	6.31
	*TRIUR3_14151*	HORVU3Hr1G085890	3.20	4.20
	*TRIUR3_19208*	HORVU5Hr1G070400	4.17	4.44
	*TRIUR3_24438*	HORVU3Hr1G053350	4.55	5.86
	*TRIUR3_29501*	HORVU5Hr1G124650	6.40	8.71
Cytochrome P450^(6)^ (CYP450)	*CYP704A2*	HORVU7Hr1G012140	5.56	7.87
	*CYP709B3*	HORVU2Hr1G027480	7.42	8.73
	*CYP72C1*	HORVU6Hr1G072990	5.62	8.71
	*CYP71A25*	HORVU7Hr1G107280	3.15	5.28
	*TRIUR3_13766*	HORVU2Hr1G109640	3.40	5.56
	*TRIUR3_23292*	HORVU7Hr1G043540	–	6.34
Glutathione-S-transferase ^(7)^	*GSTU6*	HORVU1Hr1G049190	6.01	7.61
	*GSTU7*	HORVU7Hr1G002370	–	6.01
	*GSTU8*	HORVU0Hr1G019300	5.94	7.81
	*GSTU9*	HORVU1Hr1G049270	5.43	7.55
	*GSTU18*	HORVU7Hr1G083910	7.02	6.21
Glycosyltransferase ^(8)^	*UGT73B4*	HORVU5Hr1G104580	5.68	7.67
	*UGT73B5*	HORVU5Hr1G104740	6.18	8.92
	*UGT87A2*	HORVU3Hr1G078840	5.06	6.02
	*UGT90A2*	HORVU7Hr1G042900	3.99	2.40
	*TRIUR3_03728*	HORVU2Hr1G004720	3.93	5.78
	*TRIUR3_09350*	HORVU3Hr1G023230	5.30	6.61
Lipid transfer protein	*LTPI*	HORVU3Hr1G009490	2.38	1.87
Manolyl transferase	*5MAT*	HORVU4Hr1G009300	4.60	5.73
Monooxygenase ^(11)^	*MO3*	HORVU4Hr1G072300	8.14	7.92
	*MO2*	HORVU4Hr1G072340	4.12	6.69

## Discussion

The degradation of AEs was analysed in the past mainly in sewage treatment plants^22^ and soil^16^ but not in crop species. In order to study whether metabolic or detoxifying pathways are turned on in crop plants potentially degrading AEs, which are taken up by the leaves after spraying with an agrochemical formulation, we investigated transcriptomic changes after AE application by RNA-Sequencing.

The MSD plot shows that the transcriptomic relationship between surfactant-treated barley leaves and the untreated leaves are clearly separated, which demonstrates that gene activity is responding to the surfactant treatment (Fig. 1). The response (number of DEGs) to the treatment with the two AEs is more pronounced with the polydisperse BrijL4 compared to the monodisperse C_12_E_4_ (Fig. 2a). Treatment with the polydisperse BrijL4 leads to differential regulation of 9724 genes of which ~ 50% are up and ~ 50% down-regulated (Fig. 2b). In response to the treatment with C_12_E_4_, only 6158 DEGs are differentially regulated but with a similar ratio of up and down-regulated genes. This more sensitive response in gene expression to the treatment with BrijL4 compared to C_12_E_4_ can best be explained by the chemical properties of the two AEs. BrijL4, as a typical technical polydisperse surfactant, is composed of three homologous series of alkyl chains with a varying degree of ethoxylation in each of the homologous series. The alkyl chains consist of C_10_, C_12_ and C_14_ and for each of the three chain lengths, the degree of ethoxylation varies between E_1_ to E_15_, whereas C_12_E_4_ is a 98% chemically pure (p.a.) substance^28^. This exposure to a large number of different monomers occurring in BrijL4 is sensed by the plant as a more intense stress signal leading to the modulation of about 1.6-fold more genes compared to the 98% pure C_12_E_4_ (Fig. 2).

Plant responses and adaptations to xenobiotics acting as environmental stress signals are arranged in complex metabolic networks^29^. To gain insight, into how biological processes, molecular functions and cellular compounds in barley leaves respond to AE exposure, GO terms were allocated to the DEGs and analysed for their enrichment. Moreover, MapMan was used to display DEGs in a diagram of the metabolic pathways. Our results indicate that there is a significant response to an AE treatment compared to the control treatment with water (Tables 1, 2, 3, 4). Possibly identified processes of AE degradation include the ω- and β- oxidation of the alkyl chain and the cleavage of C_2_ units from the EO unit as described in bacterial degradation^22^.

**Figure 3:**
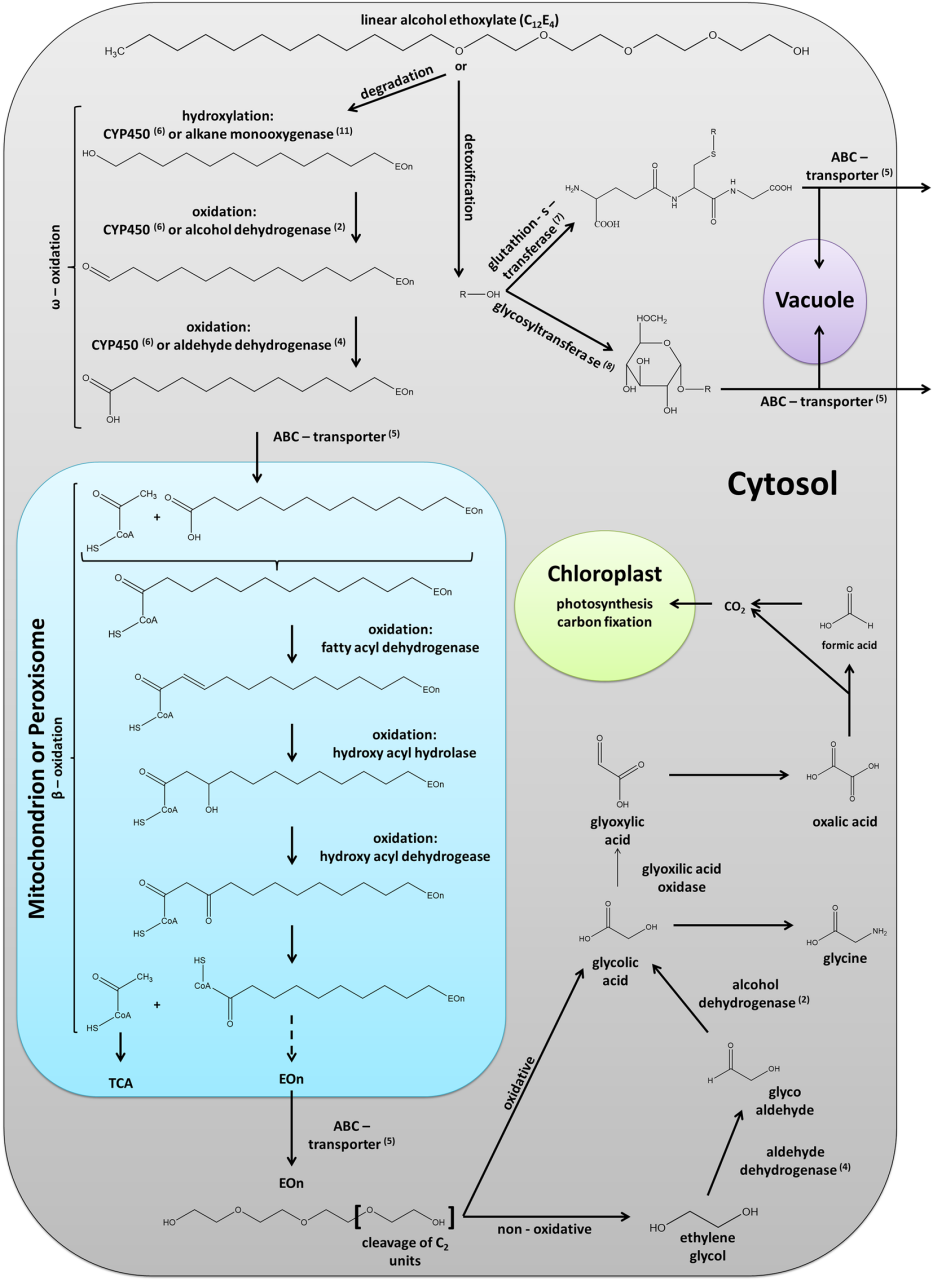
Hypothetical scheme of detoxification of the alcohol ethoxylates (AEs) deduced form from transcriptomic changes in response to surfactant treatments. Numbers in brackets next to the enzymes postulated for surfactant detoxification, indicate the corresponding genes and their differential levels of increased expression listed in Table 4. As first possible way in the degradation process, the hydroxylation of the terminal methyl group of the alkyl chain is predicted. Further steps of oxidation via the aldehyde to the carboxylic acid can take place. Carboxylated AEs could then be transported into mitochondria where they could further be degraded by β-oxidation. The two-carbon fragments released after β-oxidation could be further metabolized as acetyl Co-A by the tricarboxylic acid (TCA) cycle. As second possible way of degradation the PEG-chain of AEs could be depolymerised by the cleavage of C_2_ fragments to ethylene glycol or direct oxidation to glycolic acid. Ethylene glycol might then be further oxidised via the glycol aldehyde to glycolic acid. Glycolic acid could then further be oxidised to glyoxylic acid, oxalic acid and formic acid. In parallel glyoxylic acid could also be converted to glycine. As a third possible way, instead of degradation, detoxification of AEs by conjugation and compartmentalisation is suggested. AEs could be conjugated to glutathione or a hexose and transported into vacuoles or to the apoplast. Organelle sizes displayed in the schematic drawing are not drawn to scale.

So far, it is not clear if an initial cleavage of the alkyl chain from the PEG unit is possible in plants as it is known from some bacteria^30^. It is suggested that the process of AE detoxification forms the degradation of the hydrophobic alkyl chain by ω- and β- oxidation and the additional cleavage of C_2_ units from the PEG-unit as it was already postulated for bacteria in the past (Fig. 3)^22^. The initial step in ω-oxidation is the hydroxylation of the terminal methyl group of the alkyl chain by an alkane monooxygenase or a cytochrome P450, which then could be further oxidized via a cytochrome P450 or an alcohol dehydrogenase to an aldehyde and finally to an acid by an aldehyde dehydrogenase. Our results indicate several up-regulated cytochrome P450s and monooxygenases, which could perform these first steps of hydroxylation and oxidation (Table 4). Two alcohol dehydrogenases were also identified within the group of DEGs after the C_12_E_4_ treatment (Fig. 3; Table 4).

In addition, the aldehyde could be oxidised by an aldehyde dehydrogenase leading to the carboxylic acid, which then undergoes β-oxidation. An aldehyde dehydrogenase was also found within the DEGs of both surfactant treatments (Fig. 3; Table 4). Results with MapMan confirm that large enzyme families like oxidases and cytochromes P450, which can be involved in ω-oxidation are highly regulated in response to an AE treatment (Table 3). Analyses using Ensemble Plants showed that within the DEGs many cytochromes and other monooxygenases are up-regulated (Table 4). Moreover, in our results coenzymes such as molybdenum and iron-sulfur clusters were appearing (Table 3), which are important cofactors for cytochrome P450s and monooxygenases such as the alkane monooxygenases^31^. Up-regulated cytochrome P450s identified here, show log_2_FC changes up to 8.7 (Table 4). In parallel, cofactors such as FMA/FAD and NAD/NADP as well as acyl-carrier-proteins and acetyl transferases, which are essential for ω- and β-oxidation^32^, are highly differently expressed (Table 3). The two-carbon fragments released after β-oxidation could finally enter the tricarboxylic acid (TCA) cycle as acetyl Co-A, since the TCA cycle is strongly up-regulated after BrijL4 treatment, whereas the metabolism of Co-A metabolism is up-regulated after C_12_E_4_ treatment (Table 3).

Alternatively, to the degradation of the alkyl chain, a further starting point of the AE detoxification could be the cleavage of C_2_ fragments from the PEG-chain. This would lead to the release of either ethylene glycol or in case of a direct oxidation to glycolic acid. Ethylene glycol could then further be oxidised by an alcohol dehydrogenase via glycol aldehyde to glycolic acid by an aldehyde dehydrogenase. Glycolic acid can be further oxidised to glyoxylic acid, oxalic acid and formic acid (Fig. 3). Ethylene glycol and its oxidation products represent simple metabolites that are channelled into the general metabolic processes of cells^22^.

Tolbert and Cohan showed in 1953 with radioactively labelled glycolic acid, which was vacuum infiltrated into barley, that glycine and serine are the major products formed from glycolic acid^33^. Thus, it is very interesting that the GO terms amide biosynthetic process (GO:0043604) and cellular amino acid metabolic process (GO:0006520) are highly up-regulated, which points to a possible glycine and serine biosynthesis (Table 1). Furthermore, analysis with MapMan showed a strong regulation of photorespiration and carboxylation (Table 3). Glycolic acid has been shown to be closely associated with the CO_2_ fixation in photosynthesis and serine and glycine are important in photorespiration^33^. Moreover, the GO term organic acid metabolic process (GO:0006082) is enriched indicating that chemical reactions and pathways involving organic acids are significantly enhanced (Table 1). Further, up-regulated coenzymes are tetrahydrofolates (Table 3), which are carriers or donators of C_1_ compounds and are involved in the interconversion of glycine and serine^33^.

The enriched biological processes show clearly that due to the AE treatment protein metabolism (protein synthesis and degradation) is highly regulated as well as other biosynthetic and metabolic processes leading to the high number of differently expressed genes (Table 2). Moreover, genes involved in transport are up-regulated indicating the transport of AEs within the cell for degradation in certain compartments or the detoxification by secretion to the apoplast. The identified ABC-transporters, which are highly up-regulated, demonstrate a possible transport of AEs (Table 4). Many xenobiotics and their conjugates, which are not able to pass through a lipid membrane, are transported by drug transporters e.g. ABC-transporters^34^. Those transporters are often not highly substrate-specific and they accept a wide spectrum of structurally diverse compounds including endogenous as well as exogenous compounds^35,36^*.*

In response to the treatment with AEs, enriched activity at many membrane systems, especially the organelle membranes, mitochondria, the endoplasmatic reticulum, the Golgi apparatus, the endomembrane system and the outer membrane, occurred (Table 2). BrijL4 also shows enriched activity at the Golgi apparatus, the envelope, the endomembrane system and the outer membrane (Table 2). This supports the interpretation that especially the polydisperse BrijL4 treatment indices the detoxification of AEs by exocytosis, which seems not to be the case with the monodisperse C_12_E_4_ treatment. In both treatments, the analysis showed a small enriched molecular function of ligase, isomerise and transferase activity as well as signalling pathways such as small GTP binding, GTPase activity and catalytic activity (Table 2). Especially GTP binding and GTPase activity might be of interest in the context of regulation of vesicular trafficking in the secretory pathway between the ER and the Golgi^37^. This might facilitate the transport of AEs during degradation or detoxification. The secretory pathway can export a variety of proteins, but here AEs might be transported to the cell wall by exocytosis^38^. Hence, our results indicate a strong regulation of transport activities. This suggests that AEs, in parallel to degradation might also be detoxified by vesicular transport to sequester them in vacuoles or secret them in the apoplast^18^.

Besides the up-regulation of cytochromes P450 and monooxygenases, our results also show that acetyl transferases, methyl transferases, glutathione-S-transferases and glycosyltransferases are highly up-regulated (Table 4). Acetyl transferases may play a role in the detoxification of xenobiotics by catalysing an acetyl group to the xenobiotic. Two types of acetyl transferases might be involved. The first involves the addition of acetyl Co-A to the xenobiotic and the second involves the activation of the xenobiotic, which can then be further processed^39^. A highly up-regulated acetyl transferase was found here within the DEGs with both AE treatments (Table 4). Similar to acetyl transferases, malonyl transferases also act as acetyl-carrier-proteins and are known for their function in plant metabolism of xenobiotics ^40,41^ and with both AE treatments up-regulated manolyl transferase have also been found within the DEGs (Table 4). The addition of a methyl group to a molecule is a common transformation in the metabolism of xenobiotics and has great importance for their detoxification in plants^34^. The methylation reaction is often catalysed by S-adenosyl-L-methionine (SAM)-dependent methyl transferases. Our results indicate a high regulation of SAM as well as methyltransferases (Table 3). Moreover, SAM plays a key role in the metabolism of ethylene, a compound that might be released during the degradation of ethylene glycol, an intermediate in the degradation of the PEG-unit^42^.

In both AE treatments, glycosyl transferases and glutathione-S-transferases are also identified within the up-regulated DEGs (Table 4). Corresponding orthologous of the two glutathione-S-transferases found in the up-regulated DEGs (HORVU0Hr1G019300; HORVU7Hr1G002370) are known for their role in the detoxification of xenobiotics in *Arabidopsis*^43,44^. Those enzymes catalyze the transfer of glutathione to xenobiotics and play an important role in the transport of glutathione-tagged xenobiotics to the vacuole or the cell wall ^45^. These glutathione-conjugates might then be transported by the up-regulated ABC-Transporters (Table 4). Glycosyl transferases were also highly up-regulated within the group of DEGs (Tables 3 and 4). It is known that xenobiotics form substrates for glycosyl transferases^46^. Glycosylated of xenobiotics leads to reduced bioactivity and it enhances water solubility, which facilitates accumulation of these conjugates in the vacuole or the cell wall^47^. It is interesting that identified GO terms (Table 2) or DEGs (Tables 3 and 4), especially the glycosyl transferases, are known to be activated in response to the bacterial pathogen *Pseudomonas syringae* or *Pseudomonas fluorescence*^48–50^. Both *Pseudomonas* strains are known for their biosurfactants production. Although, those biosurfactants have different chemical structures than the AEs studied here, with both surfactants identical cellular detoxification responses might be induced.

## Conclusion

The results presented here provide a starting point for understanding the complex molecular and cellular mechanisms involved in the degradation and detoxification of AEs in crops. The enzyme families identified here, potentially acting in the degradation and/or detoxification of AEs, are in fact already quite well known to be involved in the detoxification of other xenobiotics. Our RNA-Seq study indicates that AEs could be degraded by (i) ω- and β- oxidation of the hydrophobic alkyl chain, (ii) by cleavage of C_2_ units from the polar ethoxylated part of the AEs or (iii) by sequestration of AEs into the vacuole or the apoplast (cell wall). Primarily involved in these processes are cytochromes P450, ABC transporters, acetyl- and methyl- transferases, as well as glutathione-S-transferases and glycosyltransferases. Future experiments, combining chemical-analytical and biochemical approaches, will help to directly verify in more detail, which of the potential pathways of AE detoxification and degradation suggested here, are exactly occurring in barley leaves. This knowledge could also be used in future breeding of crop plants being more resilient towards AE uptake.

## Material and methods

### Chemicals

All chemicals used were of high analytical purity (p.a.). As model surfactants, non-ionic alcohol ethoxylates were used in the experiments. The monodisperse tetraethylene glycol monododecyl ether (C_12_E_4_; Sigma-Aldrich, Germany), composed of n-dodecanol (C_12_) and 4 etyhlene oxide units (E_4_), was compared with the polydisperse BrijL4 (Sigma-Aldrich, Germany), since the calculated mean molecular weight of BrijL4 is given as C_12_E_4_. The cuticle water partition coefficient of the monodisperse C_12_E_4_, describing the lipophility of a molecule, is 6000^51^. There is no coefficient value available for the polydisperse BrijL4. However, since it’s mean chemical structure is C_12_E_4_ it can be assumed to have a similar lipophility as the monodisperse surfactant. This high lipophility of these chosen alcohol ethoxylates ensures an efficient penetration across the lipophilic cuticle inside the living leaf^24^.

### Plant material and growth conditions

Barley seeds (*Hordeum vulgare* cv. Scarlett) were originally obtained from Saatzucht Breun GmbH Co. KG (Herzogenaurach, Germany) and further propagated with permission of the breeding company at Bonn University for the experiments conducted here. Using these seeds for research was in compliance with the relevant institutional (Bonn University), national, and international guidelines and legislation. Seeds were stratified at 4 °C for one week and germinated in the dark at 25 °C on wet filter paper for 2 days. Subsequently, plants were cultivated in a growth chamber (16 h light period with 150 μmol m^-2^ s^-1^, temperature 23 °C/20 °C (day/night), relative humidity 50 to 65% (day/night)) for another 12 days on soil (Einheitserde Typ 1.5, Nitsch, Germany). Plants were watered twice a week with tap water and used for the experiments at the age of 14 days (2 days germination +12 days growth). At this stage, the 2nd leaf was fully developed and used for the experiments. In order to be consistent with our preceding study, investigating the uptake of AEs into barley leaves^24^, we have been working with exactly this plant and leaf age. Surfactants were sprayed on the leaf surfaces at aqueous concentrations of 0.1% (v/v) using an airbrush system (Start Single Action Airbrush-Pistole, Conrad, Germany). Spraying was standardized (3 × 1 s, distance to the leaf surface 10 cm) in preliminary experiments^24^, which ensured reproducible surfactant coverages of leaf surfaces of 1 µg cm^-2^. Leaves serving as controls were sprayed with tap water.

### RNA isolation and sequencing

For RNA isolation, four independent biological replicates were investigated. Two centimetre long sections from the second leaf were sampled from three individual plants and pooled to give one biological replicate. Samples were taken 8 h after the treatment with the AEs since our preceding study, investigating the uptake of AEs into barley leaves^24^, showed that all AEs had diffused into the leaves after this time period. The samples were collected in 2 ml reaction tubes with sterile steel beads inside and directly frozen in liquid nitrogen. Samples were ground with a precooled mixer mill (Retsch MM400, RETSCH GmbH, Germany) at a frequency of 30 rounds per second for one minute. Total RNA was isolated with the RNeasyPlus Universal Mini Kit (Qiagen, Venlo, Netherlands), according to the manufacturer´s instructions. RNA quality was analysed via NanoDrop (Thermo Fischer Scientific, Wilmington, Delaware, USA) and Bioanalyzer (Agilent RNA 6000 Nano Chip, Agilent Technologies: Santa Clara, USA). For all samples, a RNA integrity number (RIN) ≥ 7.7 was obtained, indicating their high quality and integrity. Library construction using polyA enrichment was done by BGI TECH SOLUTIONS. In total for each treatment and control, four biological replicates were sequenced on an Illumina HiSeq 4000sequencer (BGI TECH SOLUTIONS; Hong Kong, China).

### Processing of raw reads

Raw sequencing data of 100 bp paired-end reads were processed with CLC Genomics Workbench Version 10.0.1 (https://www.qiagenbioinformatics.com/) for further analyses. After quality trimming for low quality scores and ambiguous nucleotides, only reads with a length of more than 40 bp were retained for mapping. These reads were mapped to the barley reference genome of the genotype Morex available at Ensemble plants: Hv_IBSC_PGSB_v2, v2.36^52^ (ftp://ftp.ensemblgenomes.org/pub/plants/release-36/fasta/hordeum_vulgare/dna/) allowing large gaps of up to 50 kb to span introns. Only reads that matched unambiguously with ≥ 80% of their length and an identity of ≥ 90% to the reference genome were considered as mapped. However, by mapping reads of the genotype Scarlett to the reference Morex it cannot be decided if unmapped reads would either map to unique or multiple positions in Scarlett. Therefore, those reads, which did not map to the reference genome, were excluded from further analysis. Stacked reads, i. e. read pairs that have identical start and end coordinates and orientation, were merged into one. Subsequently, the remaining reads were mapped to the set of high confidence gene models^52^ (ftp://ftp.ensemblgenomes.org/pub/plants/release-36/gff3/hordeum_vulgare/; v2.36). Only sequences, which matched with ≥ 90% of their length and ≥ 90% similarity to this set of high confidence gene models, were considered for further analyses. Moreover, reads mapped to more than one position were excluded from subsequent analysis.

### Statistic assessment of data quality and differential gene expression

No minimum read number cut off was used for the inclusion of lowly expressed genes, enabling a more comprehensive exploration of the data. For quality control, samples were clustered in a multidimensional scaling plot (MSD plot) by using the plotMSD function implemented in the Bioconductor package *limma*^53^ in the R program (R version: 3.4.0; limma_3.32.2, https://www.r-project.org/). Distances between sample pairs were indicated as the log_2_-fold change (log_2_FC) which is defined as the estimated root-mean-square deviation for the top 500 genes with the largest standard deviation among all samples. This analysis provided a visual representation of sample relationships by spatial arrangement. Prior to further analysis, read counts have been normalized by sequencing depth and log_2_-transformed with calc-norm and TMM to meet the assumptions of a linear model. Moreover with voom, the mean–variance relationships were estimated and used to assign precision weights to each observation to adjust for heteroscedasticity^54^. To assess differences in gene expression between surfactant treatment and control, a linear model including a fixed effect for treatments was applied. The contrast fit function of the R package *limma* was used to compute pair-wise comparisons between surfactant treatment and control. To correct calculated *p*-values of the performed pairwise t-tests for multiplicity, the false discovery rate (FDR) was adjusted to ≤ 1% according to Benjamini and Hochberg^55^. Volcano plots are used to depict differentially expressed genes (DEGs) between control and treatment.

### Functional annotation of differentially expressed genes and gene ontology (GO) analysis

For postulating hypothetical pathways involved in the degradation and detoxification of AEs gene ontology (GO) categories were assigned to the differentially expressed genes. The web-based AgriGOv2.0 software (Tian et al.^27^) was used for Singular Enrichment Analysis (SEA) by comparing the list of differentially expressed genes. By filtering redundant GO terms based on their similarity^56^. MapMan (3.6.ORC1, https://mapman.gabipd.org/mapman) and REVIGO (reduce and visualize gene ontology, http://revigo.irb.hr/) were used as tools that allow displaying of large data sets in diagrams showing metabolic pathways or other processes^57^. Data were analysed and displayed in the context of pre-existing biological knowledge. Functional annotation of the differential expressed genes was done by searching putative orthologues using Ensemble Plants (https://plants.ensembl.org/index.html)^58^.

## Data Availability

The raw sequencing data have been deposited at the National Center for Biotechnology Information (NCBI) sequence read archive (https://www.ncbi.nlm.nih.gov/sra/PRJNA875119).
